# Investigations of proximity-induced superconductivity in the topological insulator Bi_2_Te_3_ by microRaman spectroscopy

**DOI:** 10.1038/s41598-021-02475-w

**Published:** 2021-11-26

**Authors:** D. Kiphart, Y. Harkavyi, K. Balin, J. Szade, B. Mróz, P. Kuświk, S. Jurga, M. Wiesner

**Affiliations:** 1 Adam Mickiewicz University, Faculty of Physics, Uniwersytetu Poznanskiego 2, 61-614 Poznan, Poland; 2 A. Chełkowski Institute of Physics and Silesian Center for Education and Interdisciplinary Research, University of Silesia, 75 Pułku Piechoty 1A, 41-500 Chorzow, Poland; 3 Institute of Molecular Physics, Polish Academy of Sciences, ul. Smoluchowskiego 17, 60-179 Poznan, Poland; 4 The NanoBioMedical Centre, Adam Mickiewicz University, Wszechnicy Piastowskiej 3, 61-614 Poznan, Poland

**Keywords:** Nanoscience and technology, Physics, Electronic devices, Materials science, Electronic properties and materials, Superconducting properties and materials, Surfaces, interfaces and thin films, Topological matter

## Abstract

We used the topological insulator (TI) Bi_2_Te_3 _and a high-temperature superconductor (HTSC) hybrid device for investigations of proximity-induced superconductivity (PS) in the TI. Application of the superconductor YBa_2_Cu_3_O_7-δ_ (YBCO) enabled us to access higher temperature and energy scales for this phenomenon. The HTSC in the hybrid device exhibits emergence of a pseudogap state for T > T_c_ that converts into a superconducting state with a reduced gap for T < T_c_. The conversion process has been reflected in Raman spectra collected from the TI. Complementary charge transport experiments revealed emergence of the proximity-induced superconducting gap in the TI and the reduced superconducting gap in the HTSC, but no signature of the pseudogap. This allowed us to conclude that Raman spectroscopy reveals formation of the pseudogap state but cannot distinguish the proximity-induced superconducting state in the TI from the superconducting state in the HTSC characterised by the reduced gap. Results of our experiments have shown that Raman spectroscopy is a complementary technique to classic charge transport experiments and is a powerful tool for investigation of the proximity-induced superconductivity in the Bi_2_Te_3_.

## Introduction

Hybrid structures of topological insulators (TI) and superconductors (SC) are an area of active interest due to the possibility of detecting Majorana modes and their potential applications in spintronics and quantum computing^1–5^. Such a hybrid device can be fabricated using classic low-temperature superconductors (LTSC) (e.g., Al, Nb) or high-temperature superconductors (HTSC) (e.g., YBa_2_Cu_3_O_7-δ_ (YBCO) or Bi_2_Sr2Ca_n−1_Cu_n_O_2n+4+δ_ (BSCCO)). Superconductivity in LTSCs is well described by the BCS theory. The nature of superconductivity of HTSCs is regarded as a non-BCS type^6,7^ and is still a matter of debate.

Investigations of devices based on LTSC (e.g., aluminium or niobium) often requires access to a sophisticated experimental setup working at temperatures much below 30 K, often at millikelvin range. Application of HTSCs is much more convenient because it enables investigations of hybrid devices at temperatures higher than the temperature of liquid nitrogen (T_LN_ = 77 K). Furthermore, high-T_c_ superconductivity at the interfaces of hybrid structures provides a wide range of parameters that can be exploited^8^. Combining semiconductors with strong-orbit coupling and HTSC with d-wave symmetry provide more stable conditions for observing Majorana bound states^9^.

Two effects are predicted to be observed when a topological insulator is brought into contact with a conventional s-wave superconductor. First is the superconducting proximity effect which induces superconducting correlations into the TI and its topologically protected surface states (TSS)^10,11^ that have unconventional p-wave symmetry^1,12^. Differences in pairing-types of the induced state and of the SC has been also reported for the hybrid Bi_2_Se_3_ and the d-wave Bi_2_Sr_2_CaCu_2_O_8+δ_ (HTSC), where the proximity-induced superconductivity showed s-wave pairing^13^. Second are proximity-induced topological states in the superconductor^10,14^. At the TI/SC interface, however, the value of the superconducting gap, Δ_sc_ in the SC is reduced to a lower value Δ_r_ and an induced gap Δ_i_ < Δ_r_ develops in the TI at temperatures lower than the critical temperature T_c_ of the superconductor^10^. The nature of the proximity-induced superconductivity (PS) is still a matter of debate, and three approaches can be distinguished. The first approach claims that the PS is induced in bulk states and can be extended to surface states^11^. The second, predicted theoretically^1^ and verified experimentally, suggests that proximity-induced superconducting states exists mainly in the surface channel^12,14,15^. The third proposes that the PS occurs due to Cooper pair tunnelling across the interface or due to the superconductor’s phonon-mediated pairing among surface electrons in the TI^16^.

The proximity-induced superconducting correlations in the TI decay on a characteristic length (called proximity length ${d}_{ind}$) from the interface. The value of this length can reach even tens of nanometres^14,16,17^. Deposition of a TI layer of thickness ${d}_{TI}\le {d}_{ind}$ on a superconducting substrate allows investigation of superconductivity and electron–phonon coupling (EPC) at the interface between the TI and the SC using charge transport experiments.

Information about processes occurring at the interface can be also obtained from Raman scattering experiments for TI layers thinner than the penetration depth of the laser beam $({d}_{TI}\le {d}_{laser})$^18^.

The light penetration depth through the TI layer is given by:

1 $${d}_{laser}= \sqrt{\pi fn{\mu }_{e}{\mu }_{m}},$$

where $f$ is the frequency of the laser, ${\mu }_{e}$ is the electron mobility, ${\mu }_{m}$ is the magnetic permeability, and $n$ is the electron concentration.

Raman scattering has been used to investigate the superconducting gap^19–22^ as well as proximity-induced superconductivity in semiconductor-superconductor hybrid structures^23–25^. Superconductivity in an investigated material is reflected in Raman spectra as changes in the frequency and linewidth of certain phonon modes as the temperature drops below T_c_.

A model based on perturbation theory^19^ relates softening and hardening of phonons in the vicinity of T_c_ with the ratio of a phonon’s energy and the energy of the superconducting gap. Phonon modes with energy greater than 2Δ_sc_ can break Cooper pairs in the superconducting state. This leads to an additional decay channel compared to the normal state and the phonon mode should exhibit hardening and broadening below T_c_. Phonon modes with energy less than 2Δ_sc_, however, cannot break the Cooper pairs. There are fewer normal state phonon-scattering events due to the gap in the density of states, so the Raman peak sharpens, and the mode softens^22,26^.

The 340, 430, and 500 cm^−1^ Raman modes of YBCO have been connected to the onset of superconductivity below T_c_.

The topological insulator Bi_2_Te_3_ belongs to the family of 3D topological insulators with a single Dirac cone^27^. Due to topologically protected surface states (TSS), charge transport in a TI is protected from scattering on non-magnetic defects and impurities^27,28^. To observe transport through TSS, the Fermi energy E_F_ of an investigated sample must be between the conduction and valence bands. This condition is usually fulfilled for very thin samples, where the thickness is between 2 and 7 quintuple layers (QLs)^29^. In thicker TI slabs, E_F_ is moved to the conduction band and transport in such samples is dominated by bulk electrons, so that conduction via TSS is difficult to detect. This also influences the formation of Cooper pairs at the interface of the TI/SC hybrid, as the position of the chemical potential affects the proximity length in the TI layer. When the chemical potential is at the Dirac point of the TI, the induced SC pairing amplitudes decay within approximately a few QLs from the interface. When the chemical potential is in the bulk conduction band, however, the SC pairing amplitudes develop away from the interface and can be attributed to coupling between the SC and bulk TI states^16^.

The gap of the d-wave superconductor YBCO has been determined to be between 16 and 25 meV^30–32^. In addition to the superconducting gap, a pseudogap has been observed at temperature, T* ≈ 203 K, i.e. above that of the critical temperature T_c_ of the superconductor^33,34^. The mechanism that leads to the pseudogap is still a matter of debate, but one proposed mechanism is due to formation of Cooper pairs above T_c_^30,35^.

The pseudogap and proximity-induced pseudogap can be investigated by both charge transport measurements and Raman spectroscopy. The emergence of the pseudogap in HTSCs was reflected in charge transport experiments as conductivity fluctuations for T > T_c_ and its value reached 50 meV^36^. The EPC in HTSC is strong enough to account for critical temperatures as high as 90 K and the coupling coefficient varies from 0.4 at 100 K to 1.4 at about 10 K^37^. Due to EPC, the pseudogap has been reflected in the Raman spectra of YBCO as a Fano-type profile of the 340 cm^−1^ Raman mode^22^. Since it has been demonstrated that both techniques can be used to detect and investigate the pseudogap in HTSCs, one can use either method to obtain reliable results.

In this paper, we present Raman light scattering investigations of the proximity induced gap at the interface of the Bi_2_Te_3_/YBCO hybrid as a result of the superconductor’s phonon mediated coupling to the TI’s electrons. Application of the high-temperature superconductor enabled optical measurements at the interface between TI/HTSC hybrid device in a wide temperature range covering both the superconducting and normal phase of the HTSC. Charge transport measurements were performed to confirm emergence of superconducting state in the investigated TI.

## Results and discussion

### Sample fabrication

**Figure 1:**
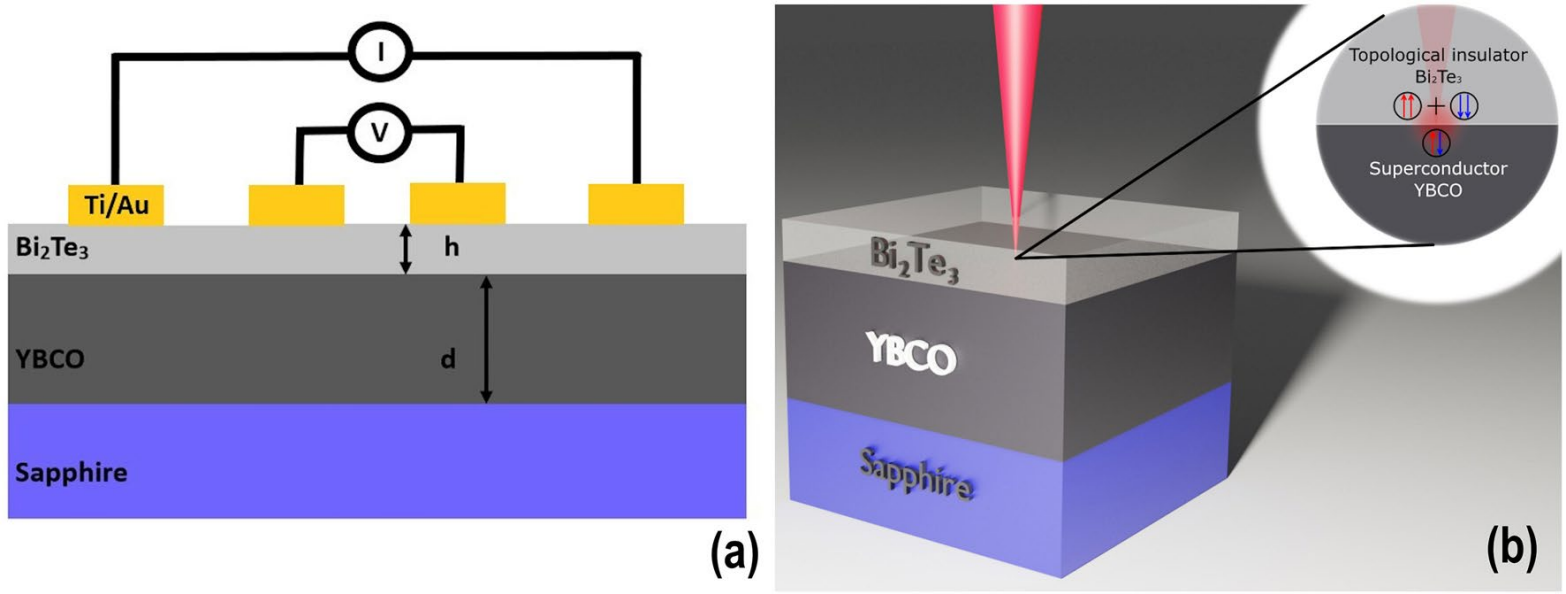
Schematic diagrams showing a cross section of the samples (not to scale) (**a**) Scheme of the charge transport measurements. The thicknesses of the films d and h for the different samples are given in Table 1. The Ti/Au electrodes for the 4-probe measurements were evaporated on top of the TI layer. (**b**) The scheme of the Raman scattering measurements showing the penetration depth of the laser beam. The inset shows the processes occurring at the TI/SC interface.

To investigate the charge and phonon transport in the TI/YBCO hybrid we used two substrates of size of 10 × 10 mm^2^ to grow the TI layer: uncoated sapphire substrate and 500 nm thick layer of the YBa_2_Cu_3_O_7_-_δ_ grown on sapphire. A 40 nm thick Bi_2_Te_3_ layer was grown on both substrates. The TI/sapphire and 500 nm thick YBCO on sapphire were used as reference samples. In total, three devices were prepared and are referred to as Sample A, Reference A, and Reference B, as summarized in Table 1 and Fig. 1a.

**Table 1:** Dimensions of reference samples and the hybrid sample used for investigations of phonon and charge transport in the Bi_2_Te_3_.

	Sapphire	YBCO (thickness, d)	Bi_2_Te_3_ (thickness, h)
Reference A	500 nm	0 nm	40 nm
Reference B	500 nm	500 nm	0 nm
Sample A	500 nm	500 nm	40 nm

The YBCO/sapphire substrate was supplied by the PI-KEM company and the uncoated sapphire by the CrysTec company. Magnetron sputtering was used to evaporate 4 nm Ti/100 nm Au electrical contacts on the top layer of the devices. Schematic diagrams of the charge transport and Raman scattering measurements are shown in Fig. 1a,b, respectively.

### Charge transport

**Figure 2:**
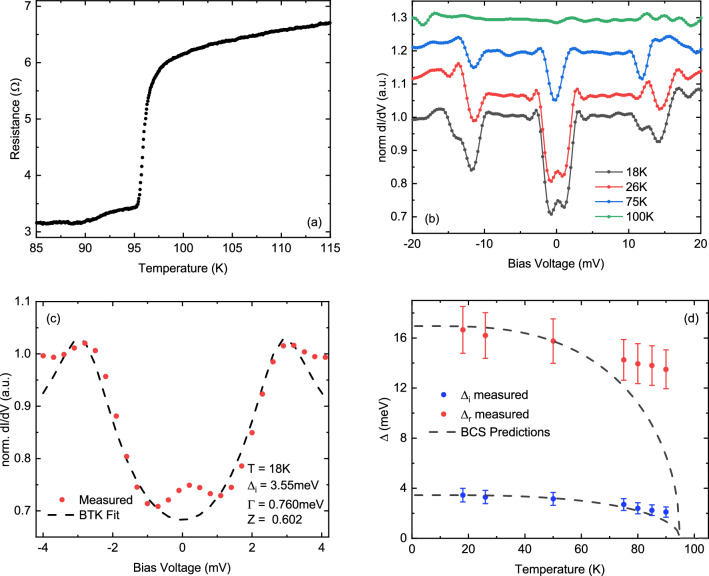
Temperature dependence of resistance of Reference B (**a**) and differential conductance of Sample A at selected temperatures (**b**). (**c**) BTK fitting of the differential conductance at 18 K with fitting parameters Δ_i_, Γ, and Z. (**d**) temperature dependencies of Δ_i_ and Δ_r_ for Sample A.

The critical temperature T_c_ of the YBCO (Reference B) was determined using 4-probe resistance measurements (Fig. 2a). The sharp drop in resistance in the Reference A sample confirms occurrence of the superconducting phase transition at T_c_ = 95 K. A finite resistance measured below the critical temperature can be attributed to the Ag paste contributing to the contact resistance of the probes as well as formation of an ultrathin oxide layer due to reaction of Ti with YBCO. The value of the superconducting gap Δ_YBCO_ = 18 meV, was determined from the bias voltage dependency of the differential conductance (dI/dV) measured at T = 18 K.

The bias voltage dependency of dI/dV for Sample A was performed at temperatures ranging from 18 to 105 K (Fig. 2b). The collected data was normalized with that obtained at T = 105 K i.e., above the T_c_ of YBCO. It reveals the emergence of a reduced gap of YBCO Δ_r_ and proximity-induced gap Δ_i_ of the TI for T < T_c_ (Fig. 2b). The differential conductance between ${\mathrm{V}}_{\mathrm{bias}}=\pm 3.6\mathrm{ mV}$ demonstrates the typical quasiparticle tunnelling effect, resulting in a conductance dip in the gapped region. A small zero-bias conductance peak (ZBCP) at ${\mathrm{V}}_{\mathrm{bias}}=0\mathrm{ V}$ however, is a signature of the Andreev reflection. The Andreev reflection (AR) is a characteristic feature for a low resistance normal metal/superconductor junction. Electrons with energy less than Δ_sc_ incident on the junction from the normal side can be transmitted as Cooper pair on the SC side, with a hole retroreflected back with opposite momentum. Alternatively, electrons with energy greater than Δ_sc_ can be transmitted. The Blonder–Tinkham–Klapwijk (BTK) model is used to calculate the current through the junction by taking these processes into account.

The value of Δ_i_ as well as quality of the junction formed by Au electrodes and the TI were determined from fitting the conductance of Sample A with the modified BTK model at 18 K, as shown in Fig. 2c. The normalized conductance, G, at low temperature can be written as^38,39^:

2 $$G= \frac{dI}{dV}=\left[1+A\left(E\right)-B(E)\right],$$

where $A\left(E\right)$ and $B\left(E\right)$ are the probabilities of Andreev and normal reflections of electrons at the interface, respectively. The terms $A\left(E\right)$ and $B\left(E\right)$ can written in terms of three parameters: the superconducting gap Δ, the quasiparticle decay rate Γ, and the interface barrier Z^40^. The details are given in the Supplementary Information.

As a result of the Andreev reflection, a small ZBCP at ${\mathrm{V}}_{\mathrm{bias}}=0\mathrm{V}$ is observed. At ultralow temperatures its value should be larger when compared to the conductance measured at energies larger that the superconducting gap. In our case, experiments were made at relatively high temperatures (T > 18 K), which resulted in a smeared ZBCP. This can be understood within the BTK theory as a reduction of the barrier strength Z due to thermally activated transport across the interface (see Supplementary Fig. S1)^41^.

In Fig. 2d the values obtained from the fitting were Δ_i_ = 3.55 meV, Γ = 0.760 meV, and Z = 0.602. For this energy, the probability of emergence of Andreev reflection was A = 0.24 whereas the probability of normal reflection was B = 0.20. The transmission probability is [1 − (A + B)]. The relation A > B confirms the superconducting properties of the TI.

The transmission coefficient T of the interface is related to Z by T = 1/(1 + Z^2^), which gives an interface transparency of 0.73. The value of Z indicates that the sample has a moderate interface transparency^39^. The broadening term Γ is a measure of the quasiparticle decay rate due to inelastic scattering processes near the interface at finite temperature. The broadening parameter can also account for a distribution of gap values for anisotropic superconductors^39^. Reasonable values of Γ at low-temperature should be proportional to the gap size, ideally with a ratio Γ/Δ ≤ 0.5^39^. The ratio for our sample is Γ/Δ = 0.21. Hybrid structures of BiSbTe_1.25_Se_1.75_^40^ and Bi_2_Se_3_^42^ on superconducting NbSe_2_ substrates have yielded similar results at low temperature, with Γ/Δ ≈ 0.27 and 0.2 respectively. The conductance is in good agreement with a single gap BTK model, however it can be noted that proximity effects can also occur at the interface Ti/Au electrodes and the superconducting TI^43,44^.

This may be related with a peak in the conductivity at zero-bias and can be accounted by using a two-gap model, with a gap corresponding to Andreev reflection at the interface of normal metal (N) (electrodes) and the superconducting TI layer^44^.

According to the BCS theory, the temperature dependency of a superconducting gap Δ_sc_ in a low-temperature superconductor is described by the BCS formula^45^:

3 $${\Delta }_{\mathrm{sc}}\left(T\right)={\Delta }_{\mathrm{sc}}\left(T=0\right)\mathrm{tanh}\left(1.74\sqrt{\frac{{T}_{C}}{T}-1}\right),$$

where: T_c_ is the critical temperature. In Fig. 2d, the measured values of both the reduced and proximity-induced gaps are compared to the BCS predictions according to Eq. (3). The values of the gaps are well fitted at low temperatures but deviate from the BCS predictions at temperatures approaching T_c_. The reason is that superconductivity in HTSCs cannot be fully described by the BCS theory, which is a mean field approximation that neglects phase fluctuations. Such fluctuations combined with weak interlayer coupling can lead to a temperature dependency of the order parameter different from that resulting from Eq. (3)^46^. Results of our experiments revealed sharp drop of temperature dependencies of both gaps at the vicinity of T_c_ (Fig. 2d). Such a non-BCS behaviour of the Δ(T) has been reported for other hybrid devices based on HTSC^42,47,48^.

The bias dependency of the conductance curves measured for Reference A followed Ohm’s law, confirming that the dips in the conductance curves of Sample A are due to the YBCO substrate.

### Raman measurements

**Figure 3:**
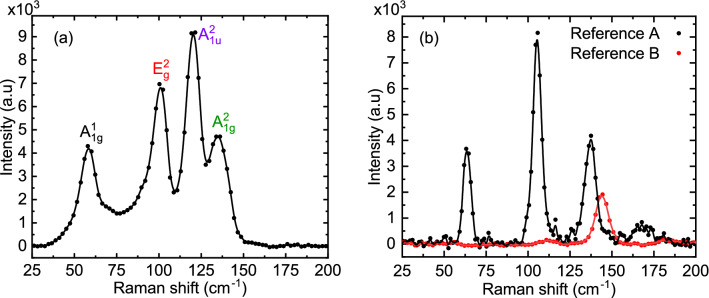
Raman spectrum for Sample A at T = 87 K with four characteristic Raman modes for Bi_2_Te_3_: A_1g_^1^, E_g_^2^, A_1g_^2^ and A_1u_^2^ (**a**). Comparison of Raman spectra of samples Reference A (black colour), and Reference B (red colour) at T = 87 K (**b**).

To distinguish the effect of the substrate, Raman light scattering experiments were performed both on reference samples (Reference A and Reference B) and on the hybrid sample (Sample A). Raman spectra collected from Sample A revealed four modes: A_1g_^1^, E_g_^2^, A_1u_^2^ and A_1g_^2^ at 61, 102, 120 and 134 cm^−1^, respectively, as shown in Fig. 3a.

The work function of Bi_2_Te_3_ is W_TI_ = 5.3 eV^18^ and of YBCO is W_YBCO_ = 6.1 eV^49^. Thus, once the materials are in contact, electron transfer is possible between the Bi_2_Te_3_ and YBCO. The infra-red (IR) active mode A_1u_^2^ (which is Raman-inactive in bulk Bi_2_Te_3_) can be seen because of symmetry breaking due to charge transfer between a substrate and the TI^18^. As the pseudogap develops into a superconducting gap at temperatures T < 100 K, the intensity of the A_1u_^2^ mode decreases (see the violet line in Supplementary Fig. S3). The increasing value of the superconducting gap at T < T_c_ limits the charge transfer and as a result, the intensity of the A_1u_^2^ mode drops significantly.

The Raman spectra of Reference A showed three characteristic Raman active modes for Bi_2_Te_3_: A_1g_^1^, E_g_^2^, and A_1g_^2^ at 61, 102, and 134 cm^−1^, respectively (Fig. 3b). Reference B revealed only one Raman active mode at 140 cm^−1^ of A_g_ symmetry^50,51^ within the Raman shift range 60–145 cm^−1^ (Fig. 3a). Therefore, the peaks at 61, 102, 120 and 134 cm^−1^ can be identified as characteristic modes for the Bi_2_Te_3_. Small differences in the Raman shifts between our samples are a result of strain due to mismatching lattice constants between the TI and the different substrates (i.e., YBCO and sapphire).

The temperature dependencies of Raman shifts of the A_1g_^1^, E_g_^2^, A_1g_^2^ modes measured in Reference A are comparable with the literature and shown Supplementary Fig. S4 and Supplementary Table S1.

**Figure 4:**
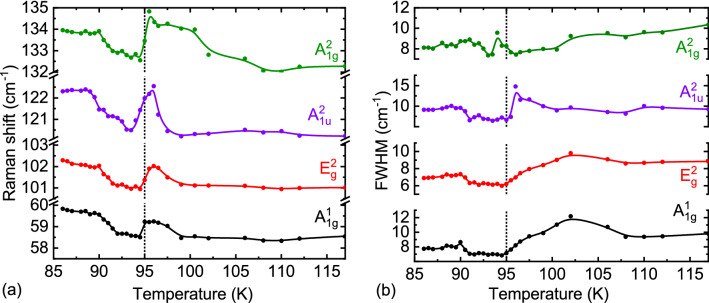
Temperature-dependencies of Raman shifts (**a**), and FWHM (**b**) of A_1g_^1^, E_g_^2^, A_1g_^2^ and A_1u_^2^ modes of Sample A.

Temperature dependencies of Raman shifts of characteristic for Bi_2_Te_3_ modes (A_1g_^1^, E_g_^2^, A_1g_^2^ and A_1u_^2^) measured on the hybrid Sample A are not linear (Fig. 4a). At temperature range 96 K < T < 100 K Raman shifts of the modes increased. A similar temperature dependency is also seen in the FWHM of the Raman modes (Fig. 4b). The modes exhibit a narrowing in the temperature range 90 K < T < 96 K for the A_1g_^2^ and A_1u_^2^ modes and 90 K < T < 102 K for the E_g_^2^ and A_1g_^1^ modes. It can be attributed to emergence of a proximity-induced pseudogap in the TI corresponding to the pseudogap of the YBCO. Such hardening of the Raman modes implies that the energy of the pseudogap was smaller compared to the energies of the Raman modes. Softening of the Raman modes at temperatures ranging between 96 and 95 K can be due to thermodynamic fluctuations of the superconducting order parameter at T_c_^52^.

At temperatures close to T_c_, the energies of the Raman modes of the TI are larger than Δ_i_ and Δ_r_. Consequently, Cooper pairs can be broken, which is reflected as a softening of all characteristic Raman modes for Bi_2_Te_3_ at T_c_ = 95 K. As the temperature decreases further below T_c_, the sizes of both gaps increase until they exceed the energy of the phonon modes, which is reflected as a hardening of the modes. When T < 90 K, changes in the values of Δ_i_ and Δ_r_ are small (Fig. 2d) and the temperature dependencies of the Raman shifts are saturated (Fig. 4a). Softening of these four modes is accompanied by decreasing intensities of these modes at T_c_. The intensities of the Raman modes as a function of temperature are provided in the Supplementary Fig. S3. The emergence of the gap is also reflected in the intensity of the A_1u_^2^ mode. For temperatures below 90 K, the intensities are comparable to that at T > T_c_¸ except the intensity if the A_1u_^2^ mode, which stayed very low. As temperature decreases, the intensity of this mode increases due to charge transfer between the YBCO substrate and the TI and decreases at temperatures where the pseudogap appears. The intensity of this mode reaches its lowest value below T_c_, when the superconducting gap and proximity-induced gap emerge, preventing the charge transfer.

Raman measurements made on Reference A did not reveal hardening or softening of the modes in the investigated sample (Supplementary Fig. S4).

Results of our investigations of the TI/HTSC hybrid can be the first step for improvement of electron–phonon coupling in the TI. In the bismuth based family (Bi_2_Se_3_ and Bi_2_Te_3_), the value of the electron–phonon coupling varies from 0.1 to 3^53–56^. Recent investigations of charge carrier dynamics in the vicinity of the critical temperature in iron-based high-temperature superconductors revealed a positive correlation between the strength of the EPC and the critical temperature^57,58^. Based on this information, we can set a hypothesis that in the topological insulator grown on a high-temperature superconductor one can enhance the EPC in the TI.

## Conclusions

We have used Raman spectroscopy to investigate a proximity-induced high-temperature superconducting phase transition in the topological insulator Bi_2_Te_3_ grown on YBCO.

Differential conductance measured as a function of temperature allowed two gaps to be determined, the proximity-induced gap in Bi_2_Te_3_ and reduced gap of the YBCO. The BTK fitting for the lowest temperature indicates a moderate barrier transparency at the interface and a ratio of Γ/Δ comparable to studies of other TI/SC hybrid structures.

Temperature dependencies of both gaps revealed a sharp drop at T_c_ = 95 K resulting from fluctuations of the order parameter of the YBCO. The critical temperature T_c_ separates two phenomena in the YBCO: emergence of the pseudogap and its transformation into a superconducting gap. Results of our experiments have shown that these two processes can be transferred to the topological insulator due to the proximity effect.

At temperature range 96 K < T < 100 K, hardening of the Raman modes can be attributed to emergence of a proximity-induced pseudogap in the TI corresponding to the pseudogap of the YBCO.

Softening of the Raman modes at temperatures ranging between 96 and 95 K can be due to thermodynamic fluctuations of the superconducting order parameter at T_c_^52^. Upon further cooling below T_c_, the frequencies of the Raman modes increased again as a result of the emergence of the proximity-induced gap in the TI. Hardening of the Raman modes implies that the energy of the gap was smaller when compared to the energy of the modes. As predicted in Bakr^59^, changes of the phonon frequency are expected to be strongest when the phonon energy coincides with the energy gap of a superconductor and to decrease with increasing the separation from the gap. Results of our experiments have shown that Raman spectroscopy is a complementary technique to classic charge transport experiments and is a powerful tool for investigation of the proximity-induced superconductivity in the Bi_2_Te_3_.

## Methods

### Raman measurements

Raman spectra of all samples were collected by the Renishaw InVia Raman spectrometer with backscattering geometry. The applied laser power was 0.58 mW and the diameter of the laser beam was 0.7 μm. Notch filters were used to eliminate the Rayleigh scattered light from the important Raman signal. The scattered light from the cells was collected and directed back through the objective lens to the spectroscopic system. The long working-distance objective had a magnification of 40×. An 1800 lines/mm diffraction grating provided a spectral resolution of ~ 1 cm^−1^ for laser wavelength of 633 nm. Exposure time and number of accumulations of each Raman spectrum were 5 s and 30 times, respectively.

Raman measurements were performed using a nitrogen cryostat. The temperature of the samples has been changed from 78 to 373 K by the Linkam PE95/T95 temperature-controlled stage.

Each measurement procedure began with heating of the system up to 373 K to remove water from the sample surface and the atmosphere of the chamber. Next, the sample was cooled down to the temperature of liquid Nitrogen (78 K). Raman spectra were collected on the heating run (with rate of 0.5 K/min) from 80 to 300 K. The spectra were collected at stabilized temperatures with a stabilization time of 5 min each. Gaussian and Lorentzian mixed function was used to fit Raman spectra using Wire 3.3 software.

### Charge transport measurements

Charge transport measurements were performed in a He cryostat at temperatures ranging from 18 to 105 K. The temperature was controlled by an Oxford Instruments ITC503 temperature controller. The differential conductance was measured using a standard four-probe method and a voltage biased scheme using a Stanford Research Systems SR803 lock-in amplifier and Keithley 2450 source meter. Differential conductance curves were collected at stabilized temperatures, with a stabilization time of 5 min. Electrical contacts were made using electron beam lithography with a Ti/Au electrode evaporated on to the surface of the samples with copper wires attached using Ag paste.

## Supplementary Information

Supplementary Information.

## Data Availability

Data from Raman and charge transport measurements are available upon request to M.Wiesner, mwiesner@amu.edu.pl.
