# Supplementary material for: Investigations of proximity-induced superconductivity in the topological insulator Bi2Te3 by microRaman spectroscopy
Source: Sci Rep. 2021 Nov 26;11:22980. doi: 10.1038/s41598-021-02475-w (PMC8626455; doi:10.1038/s41598-021-02475-w)
Supplement: Supplementary file 1 — Supplementary Information. [file 41598_2021_2475_MOESM1_ESM.pdf]

Investigations of proximity-induced superconductivity in the topological insulator Bi_2Te_3 by microRaman spectroscopy

D. Kiphart[1], Y. Harkavyi[1], K. Balin[2], J. Szade[2], B. Mróz[1], P. Kuświk[3], S. Jurga[4], *M. Wiesner[1]

[1]Adam Mickiewicz University, Faculty of Physics, Uniwersytetu Poznanskiego 2, 61-614 Poznan, Poland

[2] A. Chełkowski Institute of Physics and Silesian Center for Education and Interdisciplinary Research, University of Silesia,75 Pułku Piechoty 1A, 41-500 Chorzów, Poland

[3] Institute of Molecular Physics, Polish Academy of Sciences, ul. Smoluchowskiego 17, 60-179 Poznań, Poland

[4] The NanoBioMedical Centre, Adam Mickiewicz University , Wszechnicy Piastowskiej 3, 61-614 Poznań, Poland

*corresponding.mwiesner@amu.edu.pl

Supplementary materials

S1. BTK Fitting

At the interface between normal and superconducting materials the Andreev and normal reflections takes place with probabilities $A(E)$ and $B(E)$, respectively. In case of Sample A, the interface was formed by Au electrodes and the proximity-induced superconducting TI. The size of the proximity-induced gap in Sample A was determined by fitting the differential conductance with a modified Blonder-Tinkham-Klapwijk model. For the sake of simplicity, the fitting was performed using BTK model for a 1D, isotropic s-wave symmetry. The BTK model measures the current through the junction as the contribution of Andreev and normal reflection.

The total current through the junction is given by[S1]:

$$I_{NS} = I_0 \int_{-\infty}^{\infty} [1 + A(E) - B(E)][f(E) - f(E + eV)]dE \tag{S1}$$

where $A(E)$ and $B(E)$ are the probabilities of Andreev and normal reflections of electrons at the interface, respectively and $f(E)$ is the Fermi function. The derivative with respect to the bias voltage gives the conductance of the junction. The factor I_0 is assumed to be independent of temperature and bias. When the measured conductance curve at a given temperature is normalized by the normal state conductance, the constant of proportionality reduces to 1. The normalized conductance G, can then be expressed as[S1]:

$$G = \frac{dI}{dV} = [1 + A(E) - B(E)] \tag{S2}$$

The terms $A(E)$ and $B(E)$ can be written in terms of the fitting parameters Δ, Γ, and Z, as follows [S2].

$$A(E) = \frac{u_0^2 v_0^2}{\gamma^2} \tag{S3}$$

$$B(E) = \frac{u_0^2 - v_0^2}{\gamma^2}(Z^4 + Z^2) \tag{S4}$$

where the terms u_0, v_0, and γ are:

$$\gamma = u_0^2 + (u_0^2 - v_0^2)Z^2 \tag{S5}$$

$$u_0^2 = \frac{1}{2}\left[1 + \sqrt{\frac{(E+i\Gamma)^2 - \Delta^2}{(E+i\Gamma)^2}}\right] \tag{S6}$$

$$v_0^2 = \frac{1}{2}\left[1 - \sqrt{\frac{(E+i\Gamma)^2 - \Delta^2}{(E+i\Gamma)^2}}\right] \tag{S7}$$

The Z parameter is a phenomenological parameter to measure the elastic scattering of charge carriers. It originates from dislocations, tunnelling oxide barriers, or surface irregularities. The last factor is important in our case since the YBCO layer consists of grains which are responsible for surface roughness. Moreover, as written in the main text, Z depends on temperature (Fig. S1), which has a significant effect on the Andreev reflection.

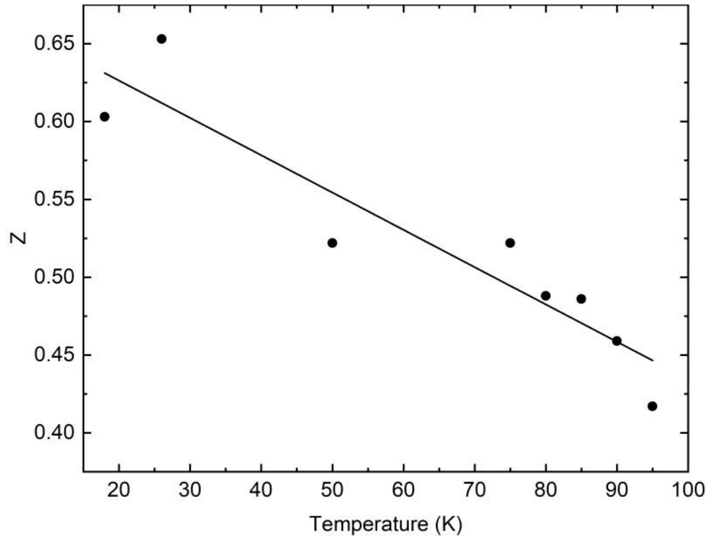

Figure S1: The temperature dependency of the Z parameter. The points are the values of Z determined from the BTK fitting and the solid line is a guide to the eye.

S2. Raman Spectra

The Raman spectra were collected for a range of temperatures from below T_c to room temperature. The original Raman spectra of the samples at 82K and 108K are shown in Figure S2.

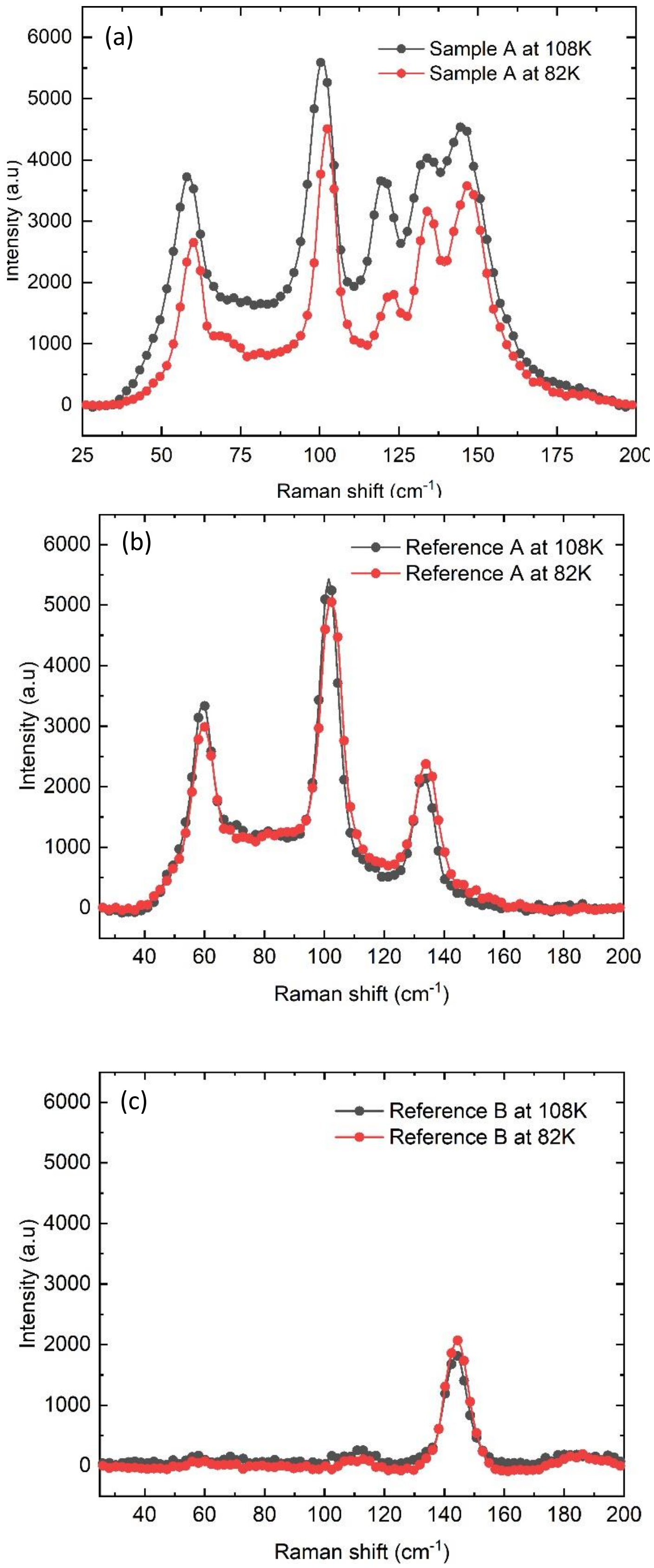

Figure S2. Raman spectra for temperatures above and below T_c of YBCO. (**a**) The Raman spectra for Sample A (hybrid sample). (**b**). The Raman spectra for Reference A (40 nm Bi_2Te_3 on sapphire substrate). (**c**). The Raman spectra for Reference B (500 nm YBCO on sapphire substrate).

Figure S3 shows the intensity of the Raman modes of Sample A as a function of the temperature. The intensities of the A_{1g}^2, E_g^2 and A_{1g}^1 modes decreased over the temperature range 91 K<T<98 K. The intensities of the Raman modes are approximately constant for temperatures outside that range. The A_{1u}^2 mode was not observed below 96 K. The intensity of the mode increased to a maximum of 10,000 a.u around 100K.

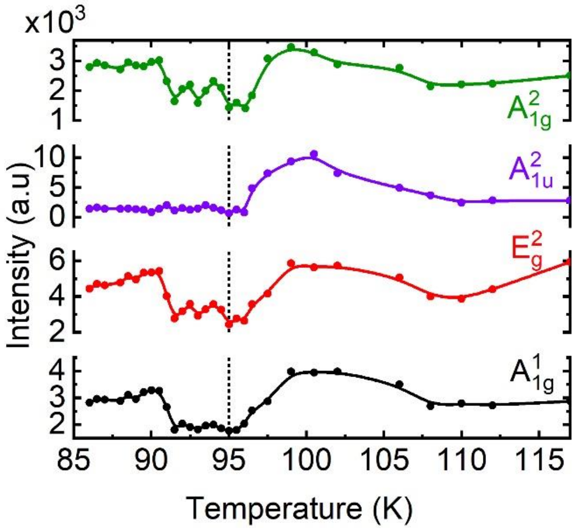

Figure S3. Temperature dependency of the intensities of the A_{1g}^1, E_g^2, A_{1g}^2 and A_{1u}^2 modes of Sample A.

To distinguish the effect of the substrate, Raman light scattering experiments were performed on both reference samples and the hybrid sample. Contrary to the hybrid sample, the Raman measurements made on Reference A (Bi_2Te_3 on sapphire substrate) did not reveal hardening or softening of the modes in the investigated sample. The temperature dependence of the Raman shift and FWHM of the A_{1g}^1, E_g^2, A_{1g}^2 modes are shown in Figure S4. The temperature dependence of the frequency of the phonon modes follows a linear relation, defined as[S3]:

$$\omega(T) = \omega_0 + \alpha T \qquad \text{(S8)}$$

where ω_0 is the frequency of the phonon mode at zero temperature and α is the first-order temperature coefficient of these phonon modes. The values of the temperature coefficients for each Raman mode were derived from a linear fitting and are listed in Table S1. It should be noted that the literature value for the A_{1g}^1 mode is for suspended nanoribbons and the presence or absence of a substrate will influence the temperature dependency of the Raman mode. Additionally, the literature values for the E_g^2 and A^2_{1g} modes are for 60nm thick Bi_2Te_3 on a SiO_2/Si substrate.

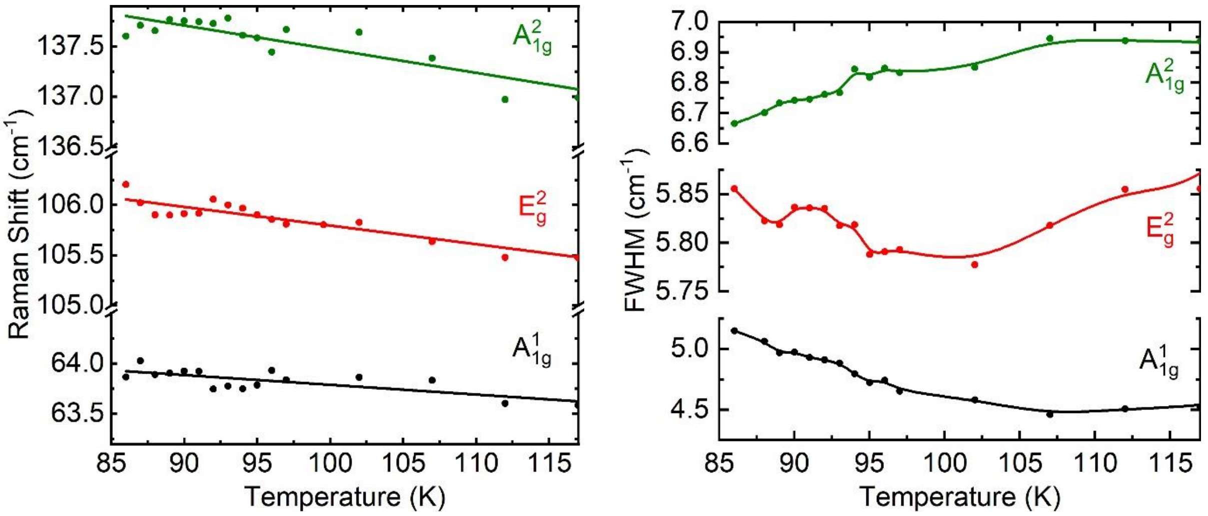

Figure S4. Temperature dependencies of A_{1g}^{1}, E_g^{2}, A_{1g}^{2} modes of Reference A.

Table S1. Temperature Coefficients (cm^{-1}/K)

Peak	Reference A	Sample A	Literature
A_{1g}^{1}	-0.00966(180)	-0.01852(184)	− 0.0158(106)[S4]
E_g^{2}	-0.01852(184)	-0.02350(283)	–0.0149[S5]
A_{1g}^{2}	-0.02350(283)	-0.00966(180)	-0.0173[S5]

References

S1. Janson, L. *et al.* Undergraduate experiment in superconductor point-contact spectroscopy with a Nb/Au junction. *Am. J. Phys.* **80**, 133–140 (2012).

S2. Banerjee, A., Sundaresh, A., Ganesan, R. & Kumar, P. S. A. Signatures of Topological Superconductivity in Bulk-Insulating Topological Insulator $BiSbTe_{1.25}Se_{1.75}$ in Proximity with Superconducting $NbSe_2$. *ACS Nano* **12**, 12665–12672 (2018).

S3. Zhou, F., Zhao, Y., Zhou, W. & Tang, D. Temperature-dependent Raman scattering of large size hexagonal Bi_2Se_3 single-crystal nanoplates. *Appl. Sci.* **8**, 1794 (2018).

S4. Park, D. *et al.* Thermal and Electrical Conduction of Single-crystal Bi_2Te_3 Nanostructures grown using a one step process. *Sci. Rep.* **6**, 13–15 (2016).

S5. Singh, M. P., Ryntathiang, S., Krishnan, S. & Nayak, P. K. Study of thermal conductivity in two-dimensional Bi_2Te_3 from micro-Raman spectroscopy. *Curr. Chinese Sci.* **01**, 1–7 (2021).
